# Spatial and multilevel determinants of delayed healthcare access following occupational accidents in Brazil

**DOI:** 10.1371/journal.pone.0360805

**Published:** 2026-10-23

**Authors:** Ahmed Fentaw Ahmed, Everton Nunes da Silva, Josivania Silva Farias, Ana Valeria Machado Mendonça, Natalia Fernandes de Andrade, Megbaru Debalkie Animut, Vanessa Resende Nogueira Cruvinel

**Affiliations:** 1 Department of Public Health, College of Medicine and Health Sciences, Injibara University, Injibara, Ethiopia; 2 University of Brasília, Brasília, Brazil; Public Library of Science, UNITED KINGDOM OF GREAT BRITAIN AND NORTHERN IRELAND

## Abstract

**Background:**

Delayed healthcare-seeking following occupational accidents increases the risk of complications, disability, and mortality. Although occupational injuries remain a major public health concern in Brazil, national evidence on the spatial distribution and multilevel determinants of Delayed healthcare-seeking is limited. This study aimed to assess the prevalence, geographic distribution, and individual and municipal-level determinants of Delayed healthcare-seeking following occupational accidents in Brazil.

**Methods:**

A nationwide cross-sectional study was conducted using 2024 data from the Brazilian Notifiable Diseases Information System. A total of 477,276 Occupational accident cases were included in the analysis. Delayed healthcare-seeking was defined as accessing healthcare services more than 24 hours after injury occurrence. A two-level mixed-effects logistic regression model was used to identify individual and municipal-level determinants, while spatial clustering techniques were applied to assess spatial clustering patterns.

**Results:**

The prevalence of Delayed healthcare-seeking was 12.2%. Significant spatial clustering was observed across Brazilian municipalities, with hotspots concentrated in the North and Midwest regions. Compared with White individuals, Black (AOR = 1.11; 95% CI: 1.06–1.15) and Brown individuals (AOR = 1.13; 95% CI: 1.10–1.16) had higher odds of Delayed healthcare-seeking. Agricultural workers were more likely to delay care (AOR = 1.20; 95% CI: 1.14–1.25). Injuries occurring in the evening showed the strongest association with delay (AOR = 3.62; 95% CI: 3.51–3.75). Higher odds of delay were also observed among individuals with lower limb injuries (AOR = 1.51), trunk/back injuries (AOR = 2.05), and multiple injuries (AOR = 1.90). Living in municipalities with lower health facility density was associated with an increased likelihood of Delayed healthcare-seeking.

**Conclusions:**

More than one in ten workers in Brazil experience Delayed healthcare-seeking following occupational injuries. Delays are associated with both social vulnerability and structural inequalities in healthcare access and exhibit significant geographic clustering. Strengthening healthcare accessibility, extending service availability, and implementing geographically targeted interventions in high-risk municipalities may improve timely care and reduce adverse occupational health outcomes. Findings highlight urgent need for geographically targeted occupational health interventions.

## Introduction

The global workforce sustains nearly 317 million occupational accidents (OA) annually, resulting in over 2.3 million deaths globally [1]. In Brazil, approximately 724,228 total work accidents were reported in 2024 [2], yielding a fatality rate of 7.4 per 100,000 workers [3]. Crucially, the clinical severity and ultimate prognosis of an OA are profoundly influenced by the speed of medical intervention; delays in seeking care are consistently associated with preventable mortality and long-term disability [4]. Beyond the human toll, OA has massive socioeconomic consequences in Brazil, costing approximately BRL 2 billion per year, nearly 4% of the national GDP without accounting for the additional diagnostic and therapeutic burdens placed on the public healthcare infrastructure [5,6].

Delay in healthcare seeking (DHS) refers to the time elapsed between the occurrence of a work-related injury and the initial presentation at a medical facility [7]. Healthcare-seeking behavior following an OA is shaped by a complex interplay of individual determinants, structural barriers, and environmental factors [4,8,9]. Literature indicates that DHS is strongly influenced by individual demographics and perceptions, including older age [10], female gender [11], and lower educational or socioeconomic status [12], alongside community-level variations such as geographic location [10,12]. According to Thaddeus and Maine’s “Three Delays Model,” acute care outcomes depend on three phases: delay in deciding to seek care (Phase 1), delay in reaching a health facility (Phase 2), and delay in receiving adequate care inside the facility (Phase 3) [7]. In a country of continental dimensions like Brazil, the first two phases are heavily exacerbated by the fragmented geographical distribution of the Unified Health System (SUS), which restricts timely access to specialized trauma services [13,14]. Consequently, workers in the North, Northeast, and rural peripheries face disproportionate physical and financial barriers to care compared to their counterparts in the highly urbanized Southeast [14].

Brazil’s occupational health landscape is characterized by persistent regional inequalities and a vast informal labor sector, both of which dictate a worker's exposure to risk and subsequent access to emergency care [15–17]. While the Information System for Notifiable Diseases (SINAN) provides robust national-level data on work-related accidents, its public reporting focuses almost exclusively on raw incidence, prevalence, and fatality rates rather than the temporal dynamics of healthcare seeking [5]. Existing national-level studies leveraging SINAN have predominantly described demographic descriptive patterns or broad epidemiological distributions of injuries [18]. However, there remains a critical limited literature utilizing advanced multilevel and spatial analysis to explicitly link individual-level accident timelines with territorial and structural inequalities. Spatial analytical methods were essential because DHS following occupational injuries is inherently influenced by geographic context, including the distribution of health facilities, transportation infrastructure, and regional socioeconomic conditions. Traditional regression models that assume independence among observations cannot adequately capture these spatial dependencies, potentially producing biased estimates and overlooking important geographic patterns. By applying spatial autocorrelation, hotspot detection, and interpolation techniques, we were able to identify whether municipalities with high DHS prevalence cluster together, detect localized high-risk areas that would otherwise remain hidden in aggregated national statistics, and generate continuous risk surfaces to guide geographically targeted interventions. This spatial approach complements the multilevel regression by explicitly accounting for geographic proximity and revealing the territorial dimension of healthcare access inequalities [13,14]. This underscores a significant evidence gap regarding why specific vulnerable populations or marginalized municipalities experience protracted delays in accessing care following an OA. Resolving this gap is essential for policymakers to transition from passive injury reporting to targeted geographically optimized occupational health interventions.

Consequently, this study aims to investigate the determinants and spatial patterns of DHS following OA in Brazil using nationwide SINAN 2024 data. To achieve this, we first estimated the national prevalence of delayed care following an OA, subsequently identifying macro level geographic patterns and significant spatial clusters of these delays across Brazilian municipalities. Finally, we evaluated the intersecting individual-level and municipal-level predictors of delayed care through an integrated spatial and multilevel analytical approach, providing a comprehensive framework to inform targeted occupational health interventions. We utilized a cross-sectional, secondary data analysis design, merging individual accident notification records from SINAN with municipal-level structural indices. Spatial autocorrelation models (Global and Local Moran’s I) were deployed to detect geographical clustering, while generalized linear mixed models (GLMM) were executed to control for nesting within municipal boundaries.

## Methods

### Study design and data source

This study employed a nationwide cross-sectional analytical design using secondary surveillance data to investigate Delayed healthcare-seeking following occupational injuries in Brazil. The analysis integrated descriptive epidemiology, multilevel regression modeling, and spatial analysis techniques to examine the prevalence, determinants, and spatial clustering of DHS.

Data were obtained from SINAN, a national health surveillance system maintained by the Brazilian Ministry of Health. SINAN compiles standardized notifications of various diseases and injuries reported by healthcare facilities nationwide, including work-related accidents

The system records detailed information on sociodemographic characteristics, occupational conditions, injury circumstances, and health service utilization, making it one of the main epidemiological surveillance systems in Brazil and widely used for monitoring notifiable conditions and supporting public health decision-making [19].

### Study population and data cleaning

Data management and cleaning procedures were conducted using Stata version 17 and Microsoft Excel. Several steps were performed to ensure data consistency and validity before analysis.

First, duplicate records were identified and removed. Cases with missing or inconsistent information related to key study variables were excluded. The study focused on individuals who received healthcare services following occupational injuries, as information on the timing of healthcare access was necessary to define the main outcome.

DHS was calculated by subtracting the time of accident occurrence from the time of healthcare attendance recorded in the SINAN database. Records with missing information on accident time, healthcare attendance time, or municipality code were excluded. In addition, records with calculated time differences that produced negative values, indicating inconsistent reporting, were removed from the dataset.

For this study, data on OA notifications reported in 2024 were downloaded from the DATASUS open data portal. The initial dataset contained 511,712 records of OA. After applying these exclusion criteria, the final analytical sample comprised 477,276 OA cases reported in Brazil in 2024.

### Inclusion and exclusion criteria

All occupational injury notifications recorded in SINAN in 2024 with complete data on the time of accident occurrence, time of first healthcare attendance, and municipality identification code were eligible for inclusion. whereas records were excluded if they were duplicates, had missing information on the outcome or key predictor variables, or presented negative calculated time differences between injury occurrence and healthcare attendance, which indicated inconsistent or erroneous reporting.

### Outcome variable

The primary outcome of interest was DHS following occupational injury. This variable was derived by calculating the time difference between the recorded time of injury occurrence and the time of first healthcare attendance. The 24-hour threshold was selected based on established evidence from injury epidemiology and trauma care literature. This cutoff is widely recognized as a critical window for timely intervention, particularly in occupational and trauma settings, where delays beyond 24 hours are consistently associated with increased risks of complications, prolonged disability, and mortality. Furthermore, the “golden hours” concept in trauma care underscores that initial medical contact within the first 24 hours significantly improves clinical outcomes and reduces the burden of long-term sequelae.

[20]. No delay: healthcare attendance within 24 hours of the injury event, and DHS: healthcare attendance more than 24 hours after injury occurrence. This binary outcome variable was generated by recording procedures performed in Stata and was used as the dependent variable in subsequent regression and spatial analyses.

### Independent variables

#### Sociodemographic characteristics.

Individual-level sociodemographic variables included: sex, age group, race/skin color, and education level. Occupational conditions were examined using variables related to employment status and occupation type: Employment situation (categorized as formal employee, informal or self-employed worker, and other employment situations) and Occupation group (categorized into manual/industrial workers, service and sales workers, agricultural workers, elementary occupations, and other occupational groups).

Variables describing injury events were also included in the analysis: Type of accident (classified as typical OA or commuting accident), Time of accident occurrence (categorized as night, morning, afternoon, or evening), and Body region injured (grouped as head/neck, upper limbs, lower limbs, trunk/back, and multiple or other body regions). To capture regional differences within Brazil, cases were classified into five geographic regions based on municipal location: North, Northeast, Southeast, South, and Midwest.

#### Municipal-level contextual variables.

To examine contextual influences on healthcare access, several municipal-level indicators were incorporated into the analysis. These indicators were obtained from official Brazilian statistical and health information systems, including the Brazilian Institute of Geography and Statistics (IBGE).

The contextual variables included: health facility density, primary healthcare coverage, municipal Gross Domestic Product (GDP), and urbanization rate. Data on municipal GDP and urbanization rate were obtained from the IBGE SIDRA database using the most recent available estimates 2022. Health facility information was extracted from the CNES database for 2023, which provides detailed records of registered healthcare establishments across Brazilian municipalities. Primary healthcare coverage indicators, reflecting the population coverage of primary healthcare teams within the Brazilian public health system, were obtained from DATASUS for 2023.

Health facility density was calculated as the number of healthcare facilities per 10,000 population, using municipal population estimates from IBGE. Expressing this indicator per 10,000 inhabitants standardizes the measure across municipalities with different population sizes and enables meaningful comparisons of healthcare service availability and infrastructure capacity [21].

All municipal-level variables were categorized into quartiles. Quartile classification divides municipalities into four groups of equal size, facilitating comparison between areas with relatively low and high levels of socioeconomic or healthcare resources while avoiding arbitrary cut-off points [22].

Municipal indicators were merged with individual-level SINAN records using the six-digit municipality identification code (CD_MUN) following standard IBGE coding procedures.

### Statistical analysis

All statistical analyses were conducted using Stata version 17.

### Descriptive analysis

Descriptive statistics were used to summarize the characteristics of OA cases. Frequencies and percentages were calculated for categorical variables to present the distribution of cases by sociodemographic, occupational, injury-related, and municipal characteristics.

### Multilevel regression analysis

To identify factors associated with delayed healthcare–seeking following OA, a two-level mixed-effects logistic regression model was fitted using the *melogit* command in Stata version 17. The multilevel approach was used to account for the hierarchical structure of the data, where individuals (level 1) were nested within municipalities (level 2).

Let *P*ᵢⱼ denote the probability of DHS for the *i*th individual in the *j*th municipality [23]. The model is specified as: $logit(P_{ij})\;=\;ln(P_{ij}\;/\;(\text{1}\;-\;P_{ij}))\;=\;\beta_{\text{0}}\;+\;{\Sigma \beta }_{k}X_{kij}\;+\;{\Sigma \gamma }_{m}Z_{mj}\;+\;u_{j}$

Where:

- β_0_ = fixed intercept (log-odds of DHS when predictors are zero)
- β_k_ = fixed-effect coefficients for individual-level variables (X_kij_)
- γ_m_ = fixed-effect coefficients for municipal-level variables (Z_mj_)
- uⱼ = random effect for municipality j, assumed to follow a normal distribution: uⱼ ~ N(0, Vₐ)

The model produces two types of effects: fixed effects, which estimate the average association between each explanatory variable and delayed care across all municipalities, and random effects, which capture unobserved heterogeneity between municipalities, essentially allowing each municipality to have its own baseline level of delayed care. Municipality identification codes were specified as the cluster-level variable, allowing the model to incorporate both individual-level and municipal-level contextual factors while accounting for potential correlation among individuals within the same municipality. Prior to fitting the multilevel models, the degree of between-municipality variability in DHS was assessed by estimating the intra-class correlation coefficient ($ICC\;=\;V_{a}\;/\;(V_{a}\;+\;3.29$)). Where: Vₐ = between-municipality variance and 3.29 = variance of the standard logistic distribution (π²/3)

The MOR represents the median increase in odds of DHS when comparing two randomly selected municipalities. $MOR\;=\;exp(\surd (\text{2}\;\times \;V_{a})\;\times \;\text{0}.\text{6}\text{7}\text{4}\text{5})$. Proportional Change in Variance (PCV) indicates the proportion of between-municipality variance explained by the included variables. $PCV\;=\;(V_{a\text{1}}\;-\;V_{a\text{2}})\;/\;V_{a\text{1}}.\;$ Where V_a1_ = variance in the empty model and V_a2_ = variance in the adjusted model [24].

Initially, bivariate analyses were conducted to examine the association between each explanatory variable and DHS. Variables with a p-value of < 0.20 in the bivariate analysis were considered candidates for inclusion in the multivariable multilevel model to avoid excluding potentially relevant predictors.

Multicollinearity among explanatory variables was assessed using the Variance Inflation Factor (VIF). Variables exhibiting high multicollinearity were carefully evaluated before inclusion in the final model to ensure the stability and interpretability of the regression estimates.

The final multivariable multilevel logistic regression model simultaneously included four models: Model 1: Empty (null) model, Model 2: Adjusted for individual-level variables, Model 3: Adjusted for municipal-level variables, and Model 4: Adjusted for both individual- and municipal-level variables

Model fit was assessed using the log-likelihood ratio (LLR) test and Akaike Information Criterion (AIC). A lower AIC and a significant LLR test indicated better model fit. Adjusted odds ratios (AOR) with corresponding 95% confidence intervals (95% CI) were reported to quantify the strength of association between explanatory variables and DHS. Statistical significance was determined at a two-sided p-value < 0.05.

### Spatial analysis

Spatial analysis was conducted to investigate the geographic distribution and spatial clustering of DHS following occupational injuries across Brazilian municipalities. Geographic analyses were performed using ArcGIS 10.8. The municipal boundary shapefiles were obtained from the IBGE. Individual case records were aggregated at the municipal level using the municipality identification code (CD_MUN) and subsequently joined to the spatial dataset. For each municipality, the proportion of DHS was calculated as the number of delayed cases divided by the total number of OA cases reported in that municipality. The spatial analyses were conducted under the assumption of spatial dependence, which was confirmed by the significant Global Moran's I statistic

### Spatial autocorrelation

Global spatial autocorrelation was assessed using the Global Moran’s I statistic to determine whether DHS was spatially clustered, dispersed, or randomly distributed across municipalities. Moran’s I values range from −1 to +1, where values close to −1 indicate spatial dispersion, values around zero indicate a random spatial pattern, and values approaching +1 indicate spatial clustering. An inverse distance spatial weight matrix was applied rather than a contiguity-based approach because healthcare access barriers, such as travel distance and transportation costs, operate along a continuous spatial gradient, where nearby municipalities exert greater influence than distant ones. Contiguity matrices treat all neighbors equally regardless of distance, which inadequately captures the distance decay effect inherent in healthcare utilization patterns. Inverse distance weighting also reduces the risk of generating spatial isolates, a common limitation with contiguity matrices in regions with large or irregularly shaped administrative units.

The analysis yielded a Moran’s I value of 0.0138, with a Z-score of 11.32 and p < 0.001, indicating a statistically significant spatial clustering of DHS across Brazilian municipalities.

### Hotspot analysis

To further explore local spatial clustering patterns, the Getis–Ord Gi* hotspot analysis was performed to identify statistically significant clusters of municipalities with high and low proportions of DHS. In this analysis, hotspot areas are municipalities with DHS that are significantly higher than expected compared with neighboring municipalities, whereas cold spots are municipalities with significantly lower proportions of delay.

Statistical significance of hotspots and cold spots was evaluated using Z-scores and corresponding p-values. Municipalities with Z-scores greater than 1.96 and p < 0.05 were considered statistically significant clusters of high values (hotspots), while municipalities with negative Z-scores of similar magnitude were considered significant cold spots.

### Spatial interpolation

Spatial interpolation techniques were applied to visualize the continuous geographic distribution of DHS across Brazil. Interpolation methods estimate values at unsampled locations using data from surrounding municipalities, allowing the identification of spatial gradients and regional patterns that may not be apparent when using discrete administrative boundaries alone.

### Ethics statement

This study was conducted using publicly available secondary data from SINAN, which contains no personally identifiable information. In accordance with Resolution No. 510/2016 of the Brazilian National Health Council, studies based on publicly accessible, anonymized data are exempt from review by a Research Ethics Committee.

## Results

### Sociodemographic characteristics

A total of 477,276 OA cases were included in the analysis. Most cases occurred among men (75.2%). The majority of individuals had secondary education (63.2%), followed by primary education (27.5%), whereas those with higher education represented 8.8% of cases, and illiterate individuals accounted for a very small proportion (0.5%). In terms of race/color, White (48.6%) and Brown (42.2%) individuals constituted the largest share of injury cases, followed by Black individuals (7.9%) and other racial groups (1.3%). The age distribution showed that occupational injuries were concentrated among young and middle-aged adults, particularly those aged 20–29 years (30.4%) and 30–39 years (25.1%), with smaller proportions observed in older age groups. Table 1

**Table 1:** Sociodemographic characteristics of OA cases and DHS in Brazil, 2024.

Variable (n)	Category	n (%)
Sex (n = 477,276)	Female	118,320 (24.8)
	Male	358,956 (75.2)
Age group (n = 474,624)	<20	24,005 (5.1)
	20–29	144,360 (30.4)
	30–39	119,348 (25.1)
	40–49	100,082 (21.1)
	50–59	61,738 (13.0)
	≥60	25,091 (5.3)
Education (n = 336,516)	Illiterate	1,770 (0.5)
	Primary	92,497 (27.5)
	Secondary	212,749 (63.2)
	Higher	29,500 (8.8)
Race/color (n = 437,265)	White	212,643 (48.6)
	Black	34,756 (7.9)
	Brown	184,365 (42.2)
	Other	5,501 (1.3)

### Occupational and injury characteristics of cases

Most injured workers were formal employees (75.3%), while informal or self-employed workers accounted for 22.8%, and other employment categories represented a small proportion (1.8%). Regarding occupational groups, manual and industrial occupations comprised the largest share of injuries (43.9%), followed by service and sales (20.9%) and other occupational groups (20.4%). Agricultural workers accounted for 8.5% of cases, while elementary occupations represented 6.3%.

The majority of injuries were classified as typical OA (82.3%), whereas commuting accidents accounted for 17.7%. Regarding the time of occurrence, nearly half of the injuries occurred during the morning (46.2%), followed by the afternoon (37.8%), with fewer cases reported in the evening and night. In terms of injury site, multiple or other body regions (35.8%) and upper limb injuries (32.9%) were the most common, followed by lower limb injuries (19.5%), while trunk/back and head/neck injuries accounted for smaller proportions. Table 2

**Table 2:** Occupational and injury characteristics of cases and DHS following occupational injuries in Brazil, 2024.

Variable (N)	Category	n (%)
Employment Situation (N = 445,964)	Formal Employee	336,012 (75.3)
	Informal/Self-employed	101,887 (22.8)
	Other	8,065 (1.8)
Occupation Group (N = 470,003)	Manual/Industrial	206,474 (43.9)
	Service & Sales	98,182 (20.9)
	Agriculture	39,868 (8.5)
	Elementary	29,641 (6.3)
	Other groups	95,838 (20.4)
Injury Site (N = 476,633)	Head/Neck	39,684 (8.3)
	Upper Limbs	156,902 (32.9)
	Lower Limbs	92,854 (19.5)
	Trunk/Back	16,387 (3.4)
	Multiple/Other	170,806 (35.8)
Cause of Injury (N = 476,349)	Transport	77,047 (16.2)
	Falls	73,115 (15.3)
	Mechanical Forces	161,941 (34.0)
	Environmental/Chemical	41,084 (8.6)
	Others	123,162 (25.9)
Type of Accident (N = 464,611)	Typical occupational	382,253 (82.3)
	Commuting accident	82,358 (17.7)
Accident Period (N = 407,516)	Night	22,133 (5.4)
	Morning	188,412 (46.2)
	Afternoon	154,159 (37.8)
	Evening	42,812 (10.5)

### Municipal-level characteristics

Regionally, the largest proportion of OA cases occurred in the Southeast (42.0%), followed by the South (30.4%) and the Northeast (12.1%), while the Midwest (9.5%) and the North (6.0%) accounted for smaller shares. Regarding health system resources, more than half of the cases (57.9%) were reported in municipalities in the highest quartile of health facility density, whereas municipalities in the lower quartiles contributed relatively smaller proportions. Regarding primary healthcare coverage, the majority of cases (74.1%) were concentrated in municipalities in the lowest coverage quartile, while areas with higher coverage levels represented progressively smaller shares.

In terms of municipal economic characteristics, most cases (82.7%) occurred in municipalities classified within the highest GDP quartile, whereas those in the lowest GDP quartile accounted for only 2.3% of cases. Similarly, the distribution by urbanization level showed that 76.6% of OA cases occurred in municipalities with the highest levels of urbanization, while municipalities with the lowest levels accounted for only 4.5% of cases. Table 3

**Table 3:** Municipal-level characteristics associated with DHS among OA cases in Brazil, 2024.

Variable (N)	Category	n (%)
Region (N = 477,254)	North	28,463 (6.0)
	Northeast	57,983 (12.1)
	Southeast	200,272 (42.0)
	South	145,128 (30.4)
	Midwest	45,408 (9.5)
Health Facility Density per 10,000 (N = 477,316)	Q1 (Lowest)	52,995 (11.1)
	Q2 (Low-Medium)	64,315 (13.5)
	Q3 (Medium)	83,722 (17.5)
	Q4 (Highest)	276,284 (57.9)
Primary Care Coverage per 10,000 (N = 477,316)	Q1 (Lowest)	353,601 (74.1)
	Q2 (Low-Medium)	65,321 (13.7)
	Q3 (Medium)	34,511 (7.2)
	Q4 (Highest)	23,883 (5.0)
GDP Category (N = 477,316)	Q1 (Lowest GDP)	11,018 (2.3)
	Q2 (Low-Middle GDP)	21,860 (4.6)
	Q3 (High-Middle GDP)	49,464 (10.4)
	Q4 (Highest GDP)	394,974 (82.7)
Urbanization Level (N = 477,316)	Q1 (Lowest)	21,512 (4.5)
	Q2 (Low-Medium)	34,674 (7.3)
	Q3 (Medium-High)	55,358 (11.6)
	Q4 (Highest)	365,772 (76.6)

### Global spatial autocorrelation

The global spatial autocorrelation analysis using Moran’s I revealed a statistically significant positive spatial clustering of DHS across Brazil (Moran’s I = 0.024, z-score = 14.92, p < 0.001). Although Moran’s I value is close to zero, indicating a low magnitude of spatial dependence, the very high z-score and significant p-value confirm that the observed pattern is not random. This suggests that municipalities with similar levels of DHS tend to be located near each other, forming significant clusters, even though the overall clustering strength is relatively modest (Fig 1)**.**

**Fig 1:**
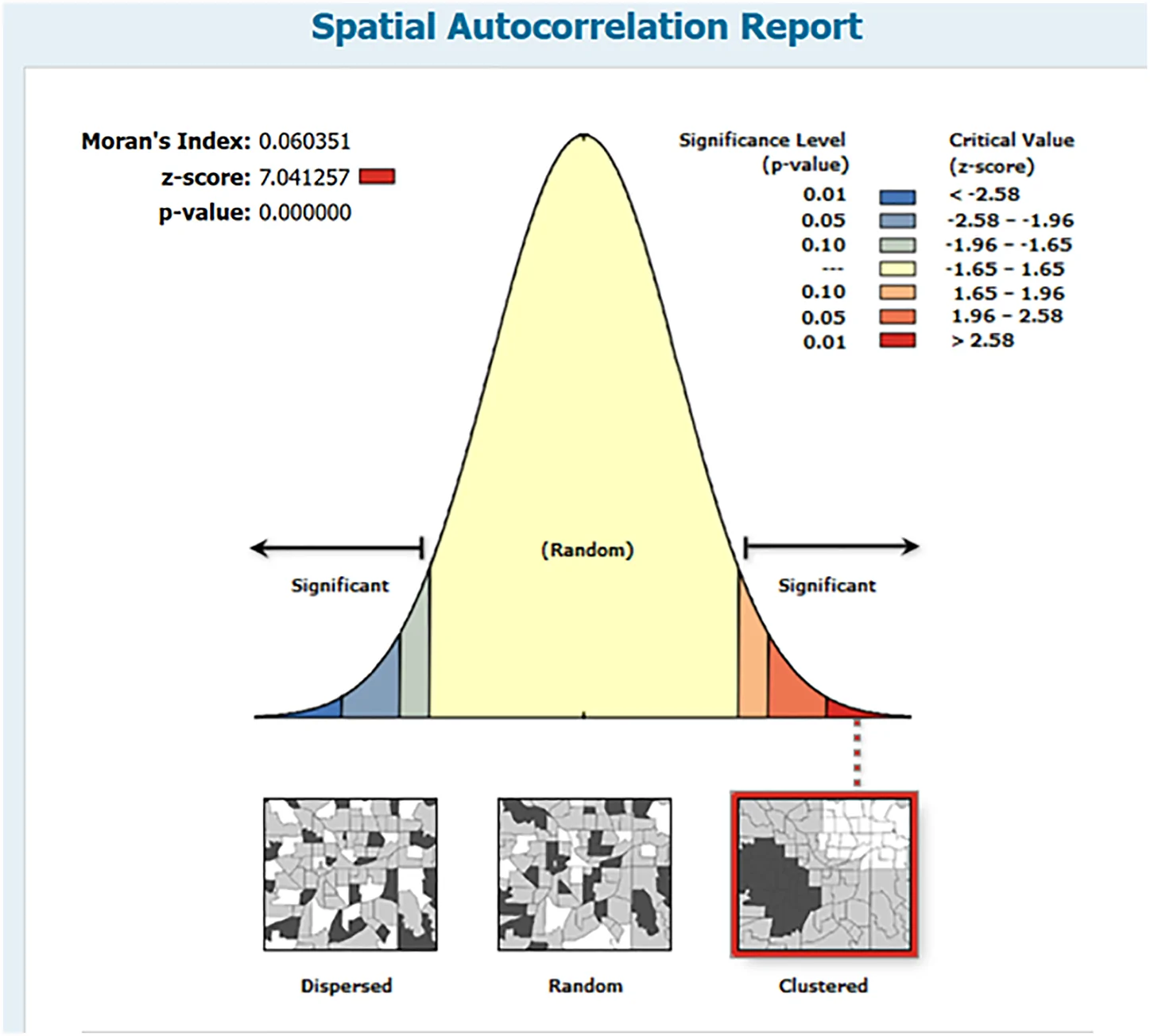
Global Spatial Autocorrelation of Delayed Healthcare Access Following Occupational Accidents in Brazil, 2024: Moran’s I Analysis.

### Spatial distribution of delayed health service access

The spatial distribution of delayed health service access after OA revealed marked geographic heterogeneity across Brazil, with clusters of high prevalence observed in multiple regions. In the Northeast region, a large concentration of municipalities with elevated delayed rates was identified, particularly in Bahia, Piauí, Maranhão, and Alagoas. Notable examples include Guajeru, Itiruçu, Abaíra, and Pilão Arcado (Bahia); Paes Landim, Rio Grande do Piauí, and Porto Alegre do Piauí (Piauí); and Santa Filomena do Maranhão, Monção, and Palmeirândia (Maranhão). Additional high-delay municipalities were observed in Feira Grande and Branquinha (Alagoas) and Gameleira (Pernambuco), indicating a broad regional pattern of limited timely healthcare access.

In the North region, municipalities such as Pau D’Arco (Pará) and Taipas do Tocantins, Novo Jardim, and Santa Terezinha do Tocantins (Tocantins) also exhibited high delayed rates.

In the Southeast region, although the pattern was less spatially clustered, several municipalities demonstrated significant delays, including Campos Gerais, Carbonita, Juramento, and Arinos (Minas Gerais); João Ramalho and Guaraçaí (São Paulo); and Cachoeiro de Itapemirim and Serra (Espírito Santo). Similarly, in the Central-West region, high-delay municipalities such as São José do Povo and Alto Paraguai (Mato Grosso) and Corumbaíba, Davinópolis, and Moiporá (Goiás) were identified, and in the South region, municipalities including Frei Rogério, Monte Carlo, and Luiz Alves (Santa Catarina) exhibited elevated delayed rates, although these were more dispersed compared to other regions(Fig 2).

**Fig 2:**
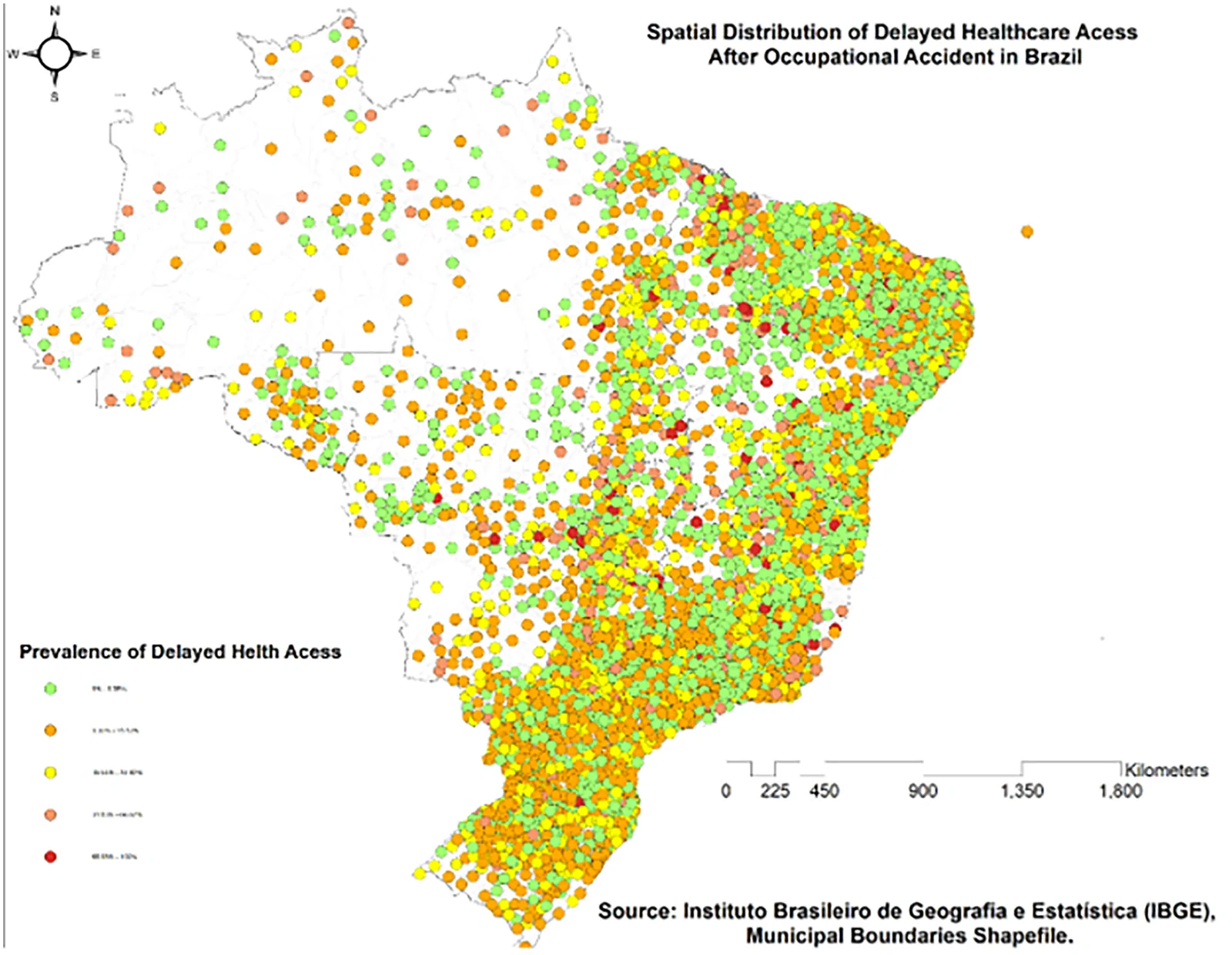
Geographic Distribution of High Delayed Healthcare Access after Occupational Injuries across Brazilian Municipalities, Brazil, 2024. Source: Authors’ analysis using official municipal boundary shapefiles from the Brazilian Institute of Geography and Statistics (IBGE).

### Getis-Ord Gi* Hotspot analysis

Getis-Ord Gi* Hotspot analysis revealed significant geographic clustering: areas with high delayed care rates (hotspots, red color) were predominantly concentrated in specific contiguous regions, indicating that DHS is not randomly distributed but rather spatially dependent. Hotspots represent municipalities with significantly higher delayed care prevalence compared to neighboring areas, whereas cold spots (low delayed care areas, blue color) were observed in other regions, where municipalities exhibited significantly lower rates of delayed healthcare utilization.

Significant hotspots (high DHS) are primarily concentrated in: the Central-West and Southeast interior (Goiás, Mato Grosso, Mato Grosso do Sul, and the Western parts of Minas Gerais), the Northern region (Parts of Amazonas, Pará, and Rondônia), and some municipalities in the Northwest and interior regions. Significant cold spots (low delayed care) are concentrated in: Southern Brazil (Rio Grande do Sul, Santa Catarina, and Parts of Paraná), Northeastern coastal region (Pernambuco, Paraíba, Rio Grande do Norte, and Ceará), and parts of the Southeast coast (São Paulo and Rio de Janeiro) (Fig 3).

**Fig 3:**
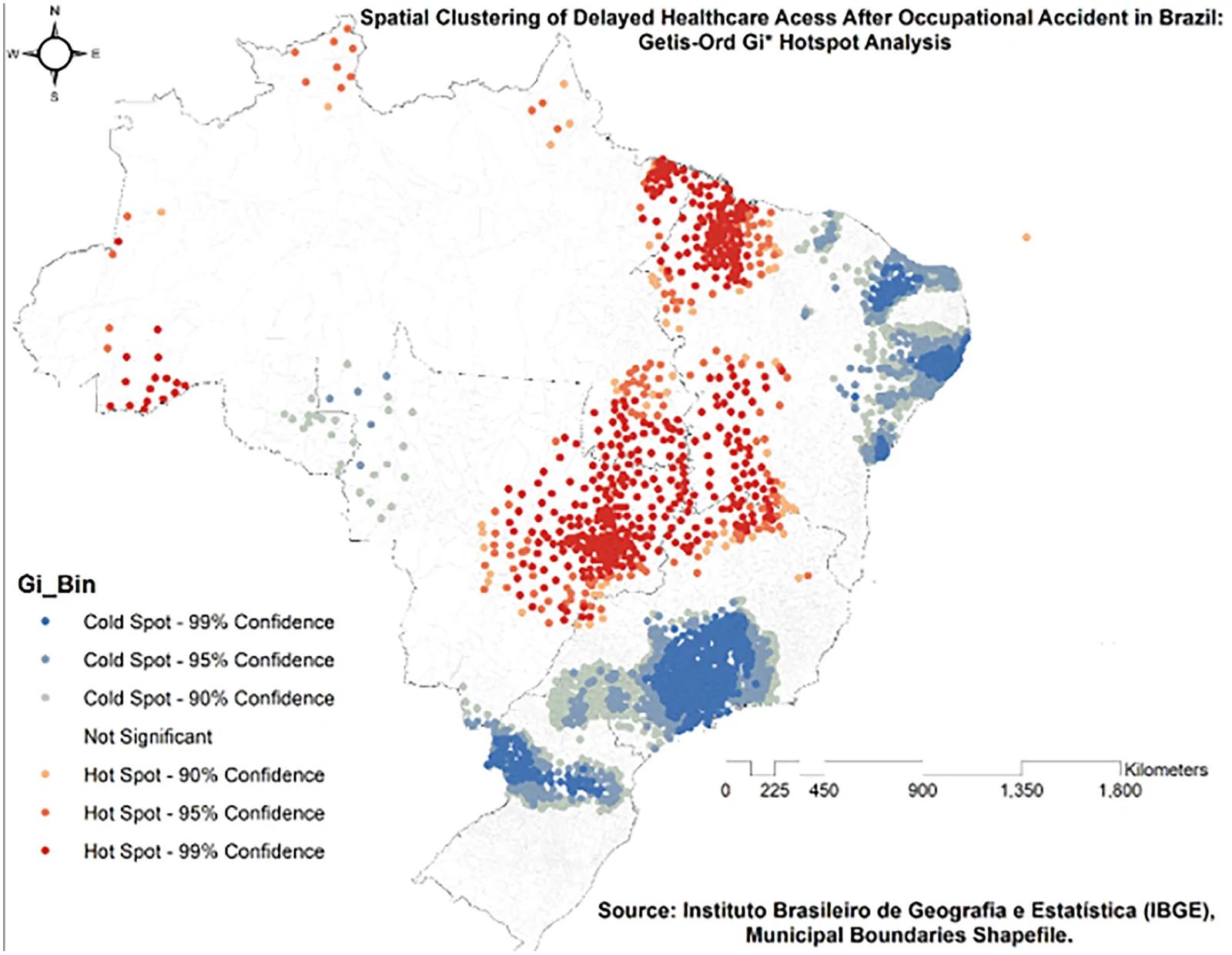
Getis-Ord Gi* Hotspot analysis of High Delayed Healthcare Access After Occupational Injuries Across Brazilian Municipalities Brazil, 2024. Source: Authors’ analysis using official municipal boundary shapefiles from the Brazilian Institute of Geography and Statistics (IBGE).

### Spatial interpolation

Spatial interpolation estimates values from non-sampled locations from known data points. The spatial interpolation analysis reveals the predicted geographic distribution of DHS following OA across Brazil, highlighting areas of varying risk intensity. Overall, the map shows that low predicted prevalence (0–24.7%) predominates across most of the country, particularly in the South, Southeast, and large portions of the Central-West, indicating relatively lower predicted levels of DHS in these regions.

However, several localized pockets of higher predicted prevalence are evident. Moderate-risk areas (24.7%–49.4%) are scattered across the country, while high-risk zones (49.4%–74.1%) and very high-risk areas (>74.1%) are concentrated in specific locations. These higher-risk pockets are mainly observed in parts of the North (Amazon region), Northeast, and selected interior areas of the Central-West and Southeast, suggesting localized disparities in healthcare access (Fig 4).

**Fig 4:**
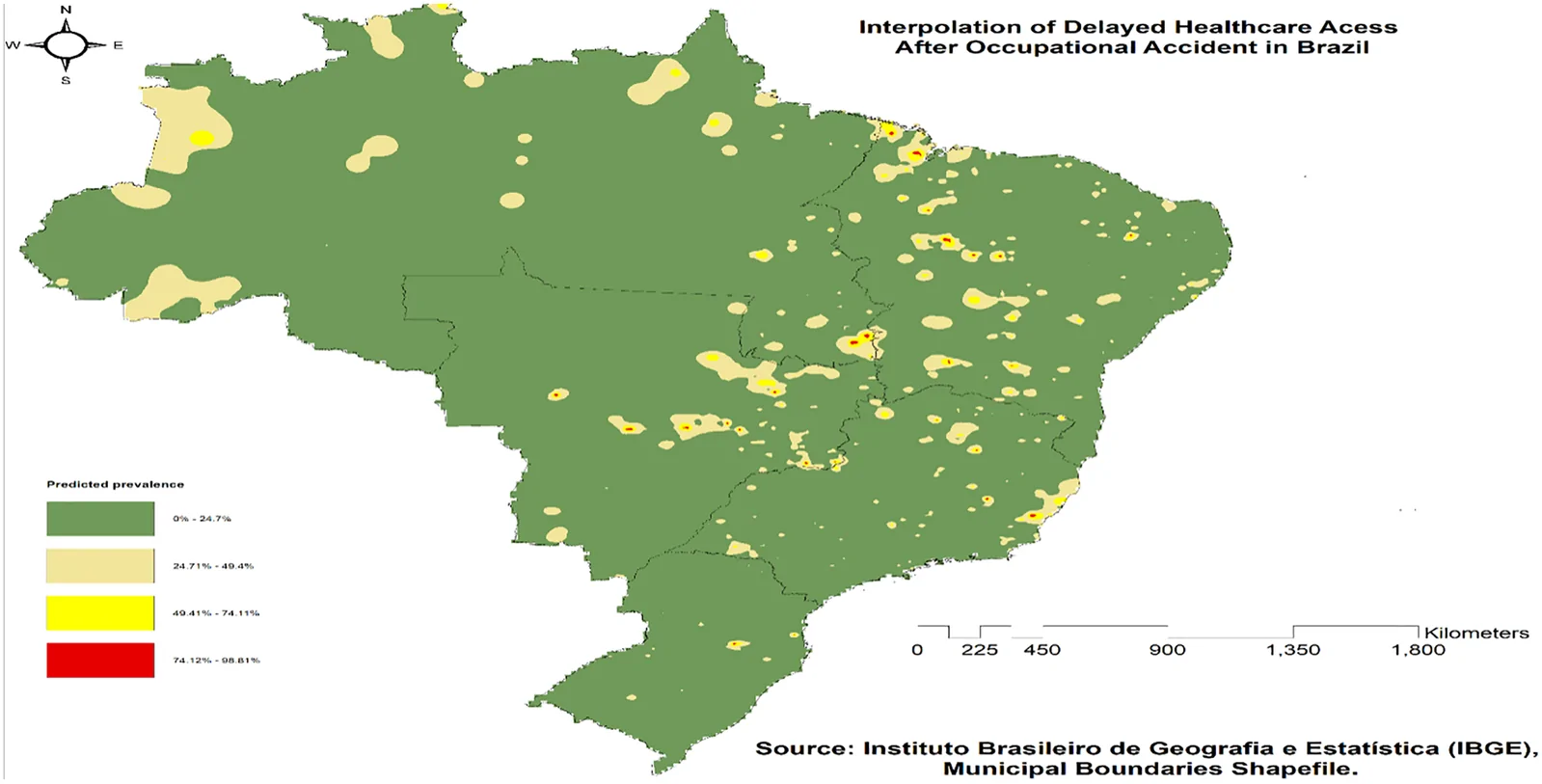
Spatial Interpolation of Predicted Prevalence of Delayed Healthcare Access Following Occupational Accidents in Brazil, 2024. Source: Authors’ analysis using official municipal boundary shapefiles from the Brazilian Institute of Geography and Statistics (IBGE).

### Factors associated with delayed health care access

This study identified several key determinants of DHS after OA. Race/color was significant predictor, with Black individuals 11% more likely [AOR = 1.11, 95% CI (1.06–1.15)] and Brown individuals 13% more likely [AOR = 1.13, 95% CI (1.10–1.16)] to experience delay compared to White individuals. Age showed a consistent gradient; individuals aged <20 years were 9% less likely to delay care [AOR = 0.91, 95% CI (0.86–0.96)], while those aged 30–39 years were 9% more likely [AOR = 1.09, 95% CI (1.05–1.12)], 40–49 years 17% more likely [AOR = 1.17, 95% CI (1.14–1.21)], 50–59 years 21% more likely [AOR = 1.21, 95% CI (1.17–1.26)], and ≥60 years 19% more likely [AOR = 1.19, 95% CI (1.13–1.26)] to delay care compared to the 20–29 age group.

Occupational characteristics were significantly associated with DHS. Individuals in “other” employment categories were 9% less likely to delay care [AOR = 0.91, 95% CI (0.82–1.00)] compared to formal employees. In terms of occupation, agricultural workers had a 20% higher likelihood of delayed care [AOR = 1.20, 95% CI (1.14–1.25)], while those in other occupational groups were 7% less likely [AOR = 0.93, 95% CI (0.90–0.96)] compared to group.

The timing of accidents emerged as the strongest predictor of DHS. Compared to morning injuries, delays were significantly higher for accidents occurring at night [AOR = 1.25, 95% CI (1.18–1.32)], afternoon [AOR = 1.95, 95% CI (1.90–2.00)], and especially evening, where the odds were more than threefold higher [AOR = 3.62, 95% CI (3.51–3.75)].

With respect to causes of injury, transport-related injuries were associated with a substantially lower likelihood of delay [AOR = 0.61, 95% CI (0.58–0.64)], while falls increased the likelihood by 12% [AOR = 1.12, 95% CI (1.08–1.16)] and environmental or chemical causes by 22% [AOR = 1.22, 95% CI (1.17–1.27)]. Injuries classified as “other causes” were also associated with a 23% lower likelihood of delay [AOR = 0.77, 95% CI (0.74–0.79)] compared to the reference category.

The body region injured was also strongly associated with delay. Head and neck injuries increased the likelihood by 6% [AOR = 1.06, 95% CI (1.01–1.11)], lower limb injuries by 51% [AOR = 1.51, 95% CI (1.46–1.56)], trunk/back injuries approximately doubled the odds [AOR = 2.05, 95% CI (1.93–2.17)], and multiple injuries increased the likelihood by 90% [AOR = 1.90, 95% CI (1.85–1.96)] compared to upper limb injuries.

The place of occurrence showed significant protective effects for non-home settings. Injuries occurring on public roads were associated with a 20% lower likelihood of delay [AOR = 0.80, 95% CI (0.77–0.83)], and injuries in other locations showed a modest reduction [AOR = 0.96, 95% CI (0.92–0.99)] compared to home injuries.

Among contextual factors, individuals residing in municipalities with the lowest health facility density were 11% more likely to delay care [AOR = 1.11, 95% CI (1.02–1.21)] compared to those in the highest quartile. Regionally, living in the Midwest was associated with an 18% higher likelihood of DHS [AOR = 1.18, 95% CI (1.07–1.31)] compared to the Southeast region. Table 4

**Table 4:** Multilevel Mixed-Effects Logistic Regression Analysis of Individual- and Municipal-Level Factors Associated with DHS Following OA in Brazil, 2024.

Variable	Category	Adjusted OR	95% CI	P-value
Sex	Female (Ref)	1.00	–	–
	Male	1.01	0.98–1.04	0.497
Race/Color	White (Ref)	1.00	–	–
	Black	1.11	1.06–1.15	<0.001
	Brown	1.13	1.10–1.16	<0.001
	Other	1.06	0.95–1.17	0.292
Age Group	20–29 (Ref)	1.00	–	–
	<20	0.91	0.86–0.96	0.001
	30–39	1.09	1.05–1.12	<0.001
	40–49	1.17	1.14–1.21	<0.001
	50–59	1.21	1.17–1.26	<0.001
	≥60	1.19	1.13–1.26	<0.001
Employment Situation	Formal (Ref)	1.00	–	–
	Informal/Self-employed	0.99	0.96–1.02	0.713
	Other	0.91	0.82–1.00	0.046
Occupation Group	Reference group (Ref)	1.00	–	–
	Service & Sales	0.98	0.95–1.01	0.297
	Agriculture	1.20	1.14–1.25	<0.001
	Elementary occupations	0.98	0.93–1.03	0.434
	Other groups	0.93	0.90–0.96	<0.001
Time of Occurrence	Morning (Ref)	1.00	–	–
	Night	1.25	1.18–1.32	<0.001
	Afternoon	1.95	1.90–2.00	<0.001
	Evening	3.62	3.51–3.75	<0.001
Cause of Injury	Reference cause (Ref)	1.00	–	–
	Transport	0.61	0.58–0.64	<0.001
	Falls	1.12	1.08–1.16	<0.001
	Environmental/Chemical	1.22	1.17–1.27	<0.001
	Others	0.77	0.74–0.79	<0.001
Body Region Injured	Upper limbs (Ref)	1.00	–	–
	Head/Neck	1.06	1.01–1.11	0.011
	Lower limbs	1.51	1.46–1.56	<0.001
	Trunk/Back	2.05	1.93–2.17	<0.001
	Multiple/Other	1.90	1.85–1.96	<0.001
Place of Occurrence	Home (Ref)	1.00	–	–
	Public road/street	0.80	0.77–0.83	<0.001
	Other location	0.96	0.92–0.99	0.023
Health Facility Density	Q4 Highest (Ref)	1.00	–	–
	Q1 Lowest	1.11	1.02–1.21	0.017
	Q2 Low-Medium	1.03	0.95–1.12	0.457
	Q3 Medium	0.94	0.87–1.01	0.111
Municipal GDP	Q4 Highest (Ref)	1.00	–	–
	Q1 Lowest	1.02	0.93–1.13	0.642
	Q2 Low-Middle	0.94	0.87–1.03	0.183
	Q3 High-Middle	0.98	0.91–1.05	0.533
Region	Southeast (Ref)	1.00	–	–
	North	1.04	0.94–1.17	0.438
	Northeast	1.01	0.92–1.09	0.898
	South	1.04	0.97–1.12	0.223
	Midwest	1.18	1.07–1.31	0.001

### Multilevel mixed-effects

The multilevel analysis demonstrates meaningful clustering of delayed care at the municipality level, as indicated by the null model ICC of 9.13% and MOR of 1.71, suggesting moderate between-municipality variation. The inclusion of individual-level factors (Model 1) and municipality-level variables (Model 2) resulted in only minimal reductions in variance (PCV = 0.88% and 0.93%, respectively), indicating that these factors alone explain a limited proportion of the observed contextual variability.

The full model (Model 3), which combines both individual and contextual factors, provided the best model fit as evidenced by the lowest AIC (227720.4) and BIC (228151.5), and achieved the highest variance reduction (PCV = 1.43%), although the ICC remained relatively stable (9.02%).

The persistence of ICC and MOR across models suggests that unmeasured municipality-level factors may still play an important role in explaining delayed care, highlighting the need for further investigation into contextual determinants. Table 5

**Table 5:** Multilevel Mixed-Effects Logistic Regression Analysis of Factors Associated with DHS Following OA.

Measure	Null model	Model 1 (individual)	Model 2 (municipality)	Model 3 (full model)
Log-likelihood	−164309.9	−113849.0	−164272.4	−113820.2
AIC	328623.8	227758.1	328568.8	227720.4
BIC	328646.0	228081.5	328701.7	228151.5
Variance (SE)	0.331 (0.015)	0.328 (0.017)	0.328 (0.015)	0.326 (0.017)
ICC	9.13%	9.06%	9.06%	9.02%
MOR	1.71	1.70	1.70	1.69
PCV (%)	Reference	0.88%	0.93%	1.43%

## Discussion

This nationwide study provides a critical, multi-layered examination of delayed health services (DHS) following OA in Brazil, revealing that post-injury delays appear to be influenced by both individual behavior and broader spatial and social inequities. By analyzing over 480,000 work-related injury notifications from SINAN, we identified a national DHS prevalence of 12.2% characterized by significant geographic heterogeneity, stark regional hotspots, and a complex interplay between individual vulnerabilities and municipal-level contextual factors that persist even after full statistical adjustment.

Our findings corroborate and significantly extend the work of Júnior & Fischer, who documented structural inequalities in the baseline incidence of workplace accidents in Brazil [14]. Moving beyond accident incidence alone, this study demonstrates that the same regions characterized by high structural vulnerability particularly the North and Midwest are precisely where injured workers face the greatest systemic barriers to receiving prompt medical attention. Spatial interpolation and hotspot analyses reveal that the risk of delay is concentrated in a contiguous “arch of occupational vulnerability” stretching from the interior of the Northeast, through the agricultural frontiers of the Midwest (Goiás, Mato Grosso), and into the Amazon region (Pará, Rondônia).

This geographic pattern maps directly onto areas where the rapid expansion of agribusiness and extractive industries has outpaced historically low investments in public health infrastructure [25]. Our finding that agricultural workers exhibit 18% higher odds of DHS (AOR = 1.20) provides robust quantitative support for this interpretation, demonstrating that a worker’s macroeconomic sector is a potent structural determinant of post-injury survival and care [26]. By delineating this specific geographic arc, this study provides a concrete spatial blueprint for targeted public health interventions rather than generic national strategies.

The high prevalence of DHS within these frontiers must be conceptualized through the classic “Three Delays Model,” which posits that maternal and trauma outcomes are shaped by delays in: (1) deciding to seek care, (2) reaching a health facility, and (3) receiving adequate treatment once inside an institution [7,27]. In a country of continental dimensions like Brazil, Phase II the delay in navigating the physical distance to a facility emerges as a critical structural bottleneck. Workers in the rural and peri-urban peripheries of the North and Midwest face formidable physical barriers, including long travel distances on poorly maintained roads and a severe deficit of reliable transportation infrastructure [28,29].

Our multilevel model provides quantitative evidence for this institutional failure, showing a persistent 10% higher likelihood of delay in municipalities with the lowest health facility density (AOR = 1.10). This underscores a vital policy reality: the universal mandate of the SUS may not ensure equitable access when physical distance and regional infrastructure are prohibitive.

In these remote territories, delayed healthcare assistance is compounded by the unequal distribution of the Mobile Emergency Care Service (SAMU 192) and specialized trauma centers, which remain heavily concentrated in developed urban centers [1,6]. In the Amazon and riverside regions, patients rely on fluvial emergency systems that are inherently slow, weather-dependent, and restricted by immense logistical limitations [3,4]. For workers injured in high-risk rural occupations such as farming, logging, or mining, these delays directly threaten the clinical “golden hour” the critical window post-trauma where rapid stabilization dictates survival and minimizes permanent disability [2,5]. Consequently, the structural deficit in regionalized emergency transit directly may contribute a treatable OA into a severe public health burden.

Beyond geography, our analysis of individual-level factors reveals how social vulnerability and structural racism are operationalized within Brazil's occupational health apparatus. The finding that Black and Brown workers experience an 11% and 13% higher likelihood of DHS, respectively, compared to White workers, serves as a stark indicator of racial inequities embedded within healthcare access. This is consistent with intersectional analyses of healthcare utilization during public health crises [18] and indicates that historical racial inequities continue to dictate real-time clinical access.

These disparities likely stem from intersecting systemic barriers, including implicit discrimination within healthcare triage, the heavy concentration of racial minorities in precarious or informal employment lacking labor protections, and residential segregation that isolates these populations from high-quality services [30,31]. Rather than treating race as a demographic variable, these findings imply that clinical equity protocols within SUS cannot be divorced from labor market realities, requiring coordinated enforcement between health authorities and labor inspectors to protect minority workforces.

This structural vulnerability is further reflected across the worker life course through a distinct age gradient. We observed a progressive increase in the odds of DHS from age 30 onward (AOR ranging from 1.09 to 1.21), contrasted with a 9% lower likelihood of delay among workers under 20. This suggests that while younger workers may navigate health networks more rapidly or report injuries immediately; increasing age is uniquely associated with structural barriers, cumulative occupational wear, or the normalization of physical pain and workplace risks over time [32–34].

Crucially, the time of injury occurrence emerged as the most powerful individual-level predictor of DHS in our model. Compared to morning injuries, the odds of delayed care escalate dramatically throughout the day, peaking sharply in the evening (AOR = 3.62), followed by the afternoon (AOR = 1.95) and night (AOR = 1.25). This pronounced “evening effect” exposes a critical temporal mismatch between working hours and healthcare availability. In many Brazilian municipalities, particularly in rural and underserved areas, primary healthcare units operate strictly on a standard daytime schedule, leaving workers injured in the late afternoon or evening without immediate local options [35]. Deferring care to the following day or attempting to navigate distant emergency services introduces substantial delays. This strong temporal pattern shifts the policy imperative from simply building more physical hospitals to altering the operational hours and shift coverage of existing primary and urgent care networks in high-risk municipal hubs [36].

Finally, the clinical characteristics and immediate environmental context of the accident dictate the speed of the care-seeking trajectory. Transport-related injuries were associated with substantially lower odds of delay (AOR = 0.61), reflecting their highly acute, visible nature, which naturally accelerates third-party bystander assistance and triggers rapid emergency response systems [34]. Conversely, injuries stemming from falls (AOR = 1.12) or environmental and chemical exposures (AOR = 1.22) significantly increase the likelihood of delay. Because these injuries are often initially perceived as manageable or lack visible trauma, workers frequently postpone care-seeking until symptoms progressively worsen [34,37,38].

This behavioral postponement is associated with the trauma. Compared to upper limb injuries, those involving the trunk or back (AOR = 2.05) and multi-site trauma (AOR = 1.90) exhibit markedly higher odds of delay, followed by lower limb injuries (AOR = 1.51). While lower limb trauma introduces a direct functional barrier to physical mobility in low-resource environments [39], trunk, back, and multi-site injuries often present with non-specific or gradually intensifying internal symptoms, leading workers to underestimate severity during initial self-assessment [40].

This diagnostic ambiguity is heavily moderated by the social architecture of the injury location. Accidental traumas occurring on public roads or streets reduce the odds of delay (AOR = 0.80) compared to those occurring within private or isolated work environments. Highly visible public settings maximize the likelihood of immediate bystander intervention, rapid injury recognition, and the swift activation of formal emergency services [34]. In contrast, injuries occurring within isolated, private, or informal workspaces are prone to delayed recognition and a heavy reliance on initial self-assessment or informal workplace remedies, which critically prolongs the time required to enter formal healthcare pipelines [41]. Taken together, these patterns demonstrate that public health interventions must actively counter the invisible nature of internal trauma and private workplace injuries through automated reporting mandates and workplace-based first-responder training.

## Conclusion

This nationwide study provides evidence that DHS following OA in Brazil is not an isolated event, but appears to be a systemic phenomenon affecting 12.2% of reported occupational injury cases included in the SINAN database, a figure that should not be generalized to all Brazilian workers, as it is limited to cases reported and captured within the surveillance system.., The findings are characterized by marked geographic and sociodemographic disparities. Empirical analysis suggests that DHS is heavily concentrated within the North and Midwest regions, with individual-level vulnerabilities appearing to be elevated among Black/Brown individuals, workers over the age of 40, those engaged in agricultural labor, and individuals sustaining injuries during evening hours. Crucially, municipal-level variations are associated with the vast majority of the observed geographic heterogeneity. This definitive finding suggests that the primary impediments to timely healthcare utilization may be structural and territorial rather than behavioral, likely rooted in the localized organization and availability of public health infrastructure.

### Recommendations

Based on these findings, this study offers five strategic recommendations for public health policy, practice, and future operational research that are directly derived from the observed associations and spatial patterns identified in this analysis:

**Extend Primary Care Operating Hours:** Public health authorities may consider expanding the operational hours of primary and urgent care clinics within high-delay municipalities, specifically targeting areas where evening shifts and nocturnal occupational activities are prevalent, as the timing of injury occurrence was the strongest individual-level predictor of DHS in this study.

**Strengthen Rural Emergency Transit:** Targeted investments may be beneficial to fortify emergency medical transport infrastructure in the North and Midwest regions, specifically through the strategic deployment of ambulances and the establishment of community-based first-responder networks, as these regions exhibited significant hotspots of delayed care and lower health facility density was associated with higher odds of delay.

**Establish Informal Sector Reporting Pathways:** Municipal and federal labor sectors may consider mandating formal, simplified reporting and care pathways for informal workers, who exhibited 20% higher adjusted odds of experiencing protracted delays in this study.

**Deploy Spatial Resource Targeting:** Financial and logistical resources could be dynamically allocated to the persistent high-risk spatial clusters identified in Goiás, Mato Grosso, Pará, and Rondônia, where unmeasured contextual elements may contribute to between-municipality disparities, as these areas were consistently identified as hotspots through Getis–Ord Gi* analysis.

**Enhance Health Information Systems:** Federal public health administrators may consider systematically strengthen the data input mechanisms of the Information System for Notifiable Diseases (SINAN). Enhancing the granularity and sensitivity of this surveillance tool could assist in better capture diverse worker profiles, formal-informal work dynamics, and exact temporal metrics, thereby generating more representative indicators to guide equitable occupational health planning.

### Strengths and limitations

This study possesses several notable strengths. Combining a multilevel and spatial analytical framework to examine the DHS following OA at a national scale in Brazil, utilizing the most recent complete year of SINAN data (2024). The large sample size (n = **477,276**) provides statistical power to detect nuanced associations and to examine heterogeneity across subpopulations. The integration of municipal-level data from IBGE and CNES enables a robust multilevel design that goes beyond purely individualistic explanations.

However, several limitations must be acknowledged. First, the cross-sectional design of the SINAN data precludes establishing temporal causality. While we identify associations, we cannot definitively determine what causes delay or whether there are other unmeasured confounders in those regions. Second, SINAN data, while comprehensive, are subject to underreporting and reporting biases. This is particularly relevant for informal workers, who may be less likely to seek formal healthcare or have their injuries notified, potentially leading to an underestimation of the true burden of DHS in this population. Additionally, regional differences in surveillance quality and completeness may influence the observed geographic patterns, as municipalities with stronger reporting systems may appear to have higher DHS prevalence simply due to more complete data capture, while areas with weaker surveillance may have underreported cases. Third, the “delay” variable, dichotomized at 24 hours, is a pragmatic but somewhat crude measure. It does not capture nuances in the *duration* of delay, which would have profound implications for morbidity and mortality. Future studies should consider examining delay as a continuous measure or using multiple thresholds (e.g., 6 hours, 12 hours, 48 hours) to better understand the clinical and epidemiological significance of different delay durations. Finally, while we used multilevel modeling to account for the hierarchical structure of the data and spatial methods to detect clustering, we did not apply spatial regression models such as spatial lag or spatial error models. Future studies could extend this work by applying geographically weighted regression (GWR) or Bayesian spatial models to further refine the estimates of contextual effects and explicitly model spatial dependence in the regression coefficients.

## Supporting information

S1 File Analytical Stata Dataset. (DTA)

S2 File Stata Analysis Code. (DO)

## Abbreviations

DHS: Delayed healthcare-seeking
OA: Occupational Accidents
SINAN: Notifiable Diseases Information System
SUS: Unified Health System
IBGE: Brazilian Institute of Geography and Statistics
CNES: National Registry of Health Establishments
DATASUS: Department of Informatics of the Brazilian Unified Health System
AOR: Adjusted Odds Ratio
MOR: Median Odds Ratio
PCV: Proportional Change in Variance
