# Supplementary material for: Spatial and multilevel determinants of delayed healthcare access following occupational accidents in Brazil
Source: PLoS One. 2026 Oct 23;21(10):e0360805. doi: 10.1371/journal.pone.0360805 (PMC13693904; doi:10.1371/journal.pone.0360805)
Supplement: Supplementary file 1 [file pone.0360805.s004.docx]

**Dear Editor and Reviewers,**

We sincerely thank you for your thorough evaluation of our manuscript and for your constructive comments and suggestions. We have carefully considered each comment and revised the manuscript accordingly. Below, we provide a detailed point-by-point response, with all changes incorporated into the revised manuscript highlighted where appropriate.

| **No.** | **Editor/Journal Comment** | **Author Response** | **Changes Made in Manuscript** |
| --- | --- | --- | --- |
| 1 | Please ensure that your manuscript meets PLOS ONE's style requirements, including those for file naming. The PLOS ONE style templates can be found at  https://journals.plos.org/plosone/s/file?id=wjVg/PLOSOne_formatting_sample_main_body.pdf and <https://journals.plos.org/plosone/s/file?id=ba62/PLOSOne_formatting_sample_title_authors_affiliations.pdf> | **Thank you for this suggestion.** The manuscript has been revised to fully comply with the PLOS ONE formatting guidelines, including file formatting, manuscript structure, and figure/table presentation. | Entire manuscript reformatted according to PLOS ONE guidelines. |
| 2 | Your ethics statement should only appear in the Methods section of your manuscript. If your ethics statement is written in any section besides the Methods, please move it to the Methods section and delete it from any other section. Please ensure that your ethics statement is included in your manuscript, as the ethics statement entered into the online submission form will not be published alongside your manuscript. | **Thank you.** The Ethics Statement has been moved exclusively to the Methods section and removed from the Data Availability/End Matter section in accordance with PLOS ONE requirements. | Methods section revised. Ethics statement removed from end matter. |
| 3 | In the online submission form, you indicated that the data that support the findings of this study are available from the corresponding author upon reasonable request.  All PLOS journals now require all data underlying the findings described in their manuscript to be freely available to other researchers, either 1. In a public repository, 2. Within the manuscript itself, or 3. Uploaded as supplementary information. This policy applies to all data except where public deposition would breach compliance with the protocol approved by your research ethics board. If your data cannot be made publicly available for ethical or legal reasons (e.g., public availability would compromise patient privacy), please explain your reasons on resubmission and your exemption request will be escalated for approval. | **Thank you for highlighting this important issue.** The Data Availability Statement has been revised. The underlying dataset and analysis files have been or uploaded as Supporting Information to ensure unrestricted access in accordance with the PLOS ONE data-sharing policy. | Data Availability Statement revised. Supporting Information uploaded. |
| 4 | [We note that Figure(s) 2, 3, 4 in your submission contain [map/satellite] images which may be copyrighted. All PLOS content is published under the Creative Commons Attribution License (CC BY 4.0), which means that the manuscript, images, and Supporting Information files will be freely available online, and any third party is permitted to access, download, copy, distribute, and use these materials in any way, even commercially, with proper attribution. For these reasons, we cannot publish previously copyrighted maps or satellite images created using proprietary data, such as Google software (Google Maps, Street View, and Earth). For more information, see our copyright guidelines: http://journals.plos.org/plosone/s/licenses-and-copyright.  We require you to either (1) present written permission from the copyright holder to publish these figures specifically under the CC BY 4.0 license, or (2) remove the figures from your submission:  a. You may seek permission from the original copyright holder of Figure(s) 2, 3, 4 to publish the content specifically under the CC BY 4.0 license.  We recommend that you contact the original copyright holder with the Content Permission Form (http://journals.plos.org/plosone/s/file?id=7c09/content-permission-form.pdf) and the following text: “I request permission for the open-access journal PLOS ONE to publish XXX under the Creative Commons Attribution License (CCAL) CC BY 4.0 (http://creativecommons.org/licenses/by/4.0/). Please be aware that this license allows unrestricted use and distribution, even commercially, by third parties. Please reply and provide explicit written permission to publish XXX under a CC BY license and complete the attached form.”  Please upload the completed Content Permission Form or other proof of granted permissions as an "Other" file with your submission.  In the figure caption of the copyrighted figure, please include the following text: “Reprinted from [ref] under a CC BY license, with permission from [name of publisher], original copyright [original copyright year].”  b. If you are unable to obtain permission from the original copyright holder to publish these figures under the CC BY 4.0 license or if the copyright holder’s requirements are incompatible with the CC BY 4.0 license, please either i) remove the figure or ii) supply a replacement figure that complies with the CC BY 4.0 license. Please check copyright information on all replacement figures and update the figure caption with source information. If applicable, please specify in the figure caption text when a figure is similar but not identical to the original image and is therefore for illustrative purposes only. The following resources for replacing copyrighted map figures may be helpful:  USGS National Map Viewer (public domain): http://viewer.nationalmap.gov/viewer/ The Gateway to Astronaut Photography of Earth (public domain): http://eol.jsc.nasa.gov/sseop/clickmap/ Maps at the CIA (public domain): https://www.cia.gov/library/publications/the-world-factbook/index.html and https://www.cia.gov/library/publications/cia-maps-publications/index.html NASA Earth Observatory (public domain): http://earthobservatory.nasa.gov/ Landsat: http://landsat.visibleearth.nasa.gov/ USGS EROS (Earth Resources Observatory and Science (EROS) Center) (public domain): http://eros.usgs.gov/# Natural Earth (public domain): http://www.naturalearthdata.com/](http://track.editorialmanager.com/CL0/We%2520note%2520that%2520Figure(s)%25202,%25203,%25204%2520in%2520your%2520submission%2520contain%2520%255bmap%2Fsatellite%255d%2520images%2520which%2520may%2520be%2520copyrighted.%2520All%2520PLOS%2520content%2520is%2520published%2520under%2520the%2520Creative%2520Commons%2520Attribution%2520License%2520(CC%2520BY%25204.0),%2520which%2520means%2520that%2520the%2520manuscript,%2520images,%2520and%2520Supporting%2520Information%2520files%2520will%2520be%2520freely%2520available%2520online,%2520and%2520any%2520third%2520party%2520is%2520permitted%2520to%2520access,%2520download,%2520copy,%2520distribute,%2520and%2520use%2520these%2520materials%2520in%2520any%2520way,%2520even%2520commercially,%2520with%2520proper%2520attribution.%2520For%2520these%2520reasons,%2520we%2520cannot%2520publish%2520previously%2520copyrighted%2520maps%2520or%2520satellite%2520images%2520created%2520using%2520proprietary%2520data,%2520such%2520as%2520Google%2520software%2520(Google%2520Maps,%2520Street%2520View,%2520and%2520Earth).%2520For%2520more%2520information,%2520see%2520our%2520copyright%2520guidelines:%2520http:%2Fjournals.plos.org%2Fplosone%2Fs%2Flicenses-and-copyright.%250b%250bWe%2520require%2520you%2520to%2520either%2520(1)%2520present%2520written%2520permission%2520from%2520the%2520copyright%2520holder%2520to%2520publish%2520these%2520figures%2520specifically%2520under%2520the%2520CC%2520BY%25204.0%2520license,%2520or%2520(2)%2520remove%2520the%2520figures%2520from%2520your%2520submission:%250b%250ba.%2520You%2520may%2520seek%2520permission%2520from%2520the%2520original%2520copyright%2520holder%2520of%2520Figure(s)%25202,%25203,%25204%2520to%2520publish%2520the%2520content%2520specifically%2520under%2520the%2520CC%2520BY%25204.0%2520license.%2520%250b%250bWe%2520recommend%2520that%2520you%2520contact%2520the%2520original%2520copyright%2520holder%2520with%2520the%2520Content%2520Permission%2520Form%2520(http:%2Fjournals.plos.org%2Fplosone%2Fs%2Ffile%3Fid=7c09%2Fcontent-permission-form.pdf)%2520and%2520the%2520following%2520t) | **Thank you.** Thank you for this observation. Figures 2–4 are original figures created by the authors using publicly available municipal boundary shapefiles from the Brazilian Institute of Geography and Statistics (IBGE). No copyrighted Google Maps, Google Earth, satellite imagery, or other proprietary basemaps were used. We have clarified the source of the geographic data in the figure captions. | Figures 2–4 captions updated. |
| 5 | Please ensure that you refer to Figure 1, 2, 3, 4 in your text as, if accepted, production will need this reference to link the reader to the figure. | **Thank you.** References to Figures 1–4 have been checked and were appropriate throughout the Results section. |  |
| 6 | Please include a separate caption for each figure in your manuscript. | **Completed.** Separate captions have been provided for all figures. | Figure captions checked and were separte. |
| 7 | If the reviewer comments include a recommendation to cite specific previously published works, please review and evaluate these publications to determine whether they are relevant and should be cited. There is no requirement to cite these works unless the editor has indicated otherwise. | **Thank you.** We carefully evaluated all available and updated references. Relevant studies have been incorporated where appropriate to strengthen the background and discussion, while references not directly relevant to our study objectives were not included. | Introduction and Discussion updated. |

**Additional Editor Comments:**

| **Editor Comment** | **Author Response** | **Changes Made** |
| --- | --- | --- |
| The statistical analysis is appropriate, although it is important to verify and report any residual spatial autocorrelation after the multilevel model. | Thank you for this valuable suggestion. We conducted an assessment of residual spatial autocorrelation using Global Moran's I on the municipality-level residuals from the final multilevel model. The results indicated no significant residual spatial autocorrelation, suggesting that the multilevel model adequately accounted for the spatial dependence in the data. Therefore, additional spatial regression models were not considered necessary for the objectives of the present study. | Methods and Results updated. |
| Clarify data availability. | Revised according to the PLOS ONE Data Availability Policy. | Data Availability Statement revised. And the date set upladed as supplementary file |
| Minor English revisions. | The manuscript has undergone thorough language editing to improve grammar, clarity, readability, and consistency. | Entire manuscript revised. |
| Delayed healthcare seeking may not reflect only individual behavior. | We agree and have revised the terminology throughout the manuscript. The outcome is now described as **"delayed healthcare access following occupational injury"** where appropriate, emphasizing that delays may reflect individual, geographic, organizational, and structural barriers rather than solely individual decisions. | Title, Abstract, Methods, Results and Discussion revised. |
| Justify the 24-hour cutoff. | Additional justification has been added based on clinical practice and previous literature. | Methods revised. |
| Strengthen justification for spatial analysis. | Expanded substantially. We now explain the rationale for investigating geographic heterogeneity, describe the spatial weights matrix, hotspot analysis, Moran's I, and assessment of residual spatial dependence. | Introduction. |
| Replace causal language. | All causal have been replaced with equivalent terminology throughout the manuscript. | Entire manuscript revised. |
| Moderate policy recommendations. | Policy implications have been revised to distinguish recommendations supported directly by the findings from broader proposals. | Discussion revised. |
| Avoid generalizing prevalence to all Brazilian workers. | The manuscript now clearly states that the estimated prevalence applies only to occupational accidents reported in SINAN. | Discussion revised. |
| Expand limitations. | Additional limitations regarding underreporting, data completeness, surveillance quality, and regional reporting differences have been included. | Limitations expanded. |
| Emphasize effect sizes rather than only statistical significance. | Results and Discussion have been revised to place greater emphasis on effect sizes, confidence intervals, and epidemiological relevance. | Results and Discussion revised. |

**Response to Reviewer #1**

| **Reviewer Comment** | **Author Response** | **Changes Made** |
| --- | --- | --- |
| The abstract is slightly dense; consider shortening methodological detail (e.g., “Global Moran’s I and Getis–Ord Gi* statistics” could be summarized as “spatial clustering techniques”). | Thank you. The abstract has been streamlined by reducing methodological detail while preserving essential findings. | Abstract revised. |
| Emphasize the policy relevance more strongly in the conclusion (e.g., “Findings highlight urgent need for geographically targeted occupational health interventions”) | Added stronger policy implications in the conclusion. | Abstract revised. |
| Some sentences are long and could be split for readability (e.g., “Beyond the human toll, OA exacts massive socioeconomic consequences…”). | Long sentences were revised for improved readability. | Introduction revised. |
| Clarify novelty: explicitly state how this study differs from prior SINAN based analyses (e.g., “Unlike previous descriptive studies, this research integrates multilevel and spatial modeling”). | A paragraph highlighting the novelty of integrating multilevel and spatial analyses has been added. | Introduction revised. |
| Consider tightening references: currently, citations (5, 6, 8, 9, 13, 15–17) are clustered; grouping them by theme would improve flow | References have been reorganized thematically. | Introduction revised. |
| The statistical model equation is well presented, but could be simplified for non technical readers (perhaps move full formula to appendix). | The statistical equation has been simplified in the main text. | Methods revised. |
| Clarify exclusion criteria: specify how many records were removed at each step (duplicates, missing values, negative time differences). | Additional details describing excluded records at each stage have been added. | Methods revised. |
| Spatial analysis: explain why inverse distance weighting was chosen over contiguity matrices. Results | A justification for selecting inverse distance weighting has been added. | Methods revised. |
| Tables 1–3 are heavy with numbers; consider adding plain language summaries (e.g., “Most injuries occurred among men in their 20s and 30s, particularly in manual/industrial occupations”). | We considered using more plain language in the result part. | Results revised. |
| Highlight effect sizes more narratively: e.g., “Evening injuries were over three times more likely to result in delayed care.” | Additional text interpreting important adjusted odds ratios has been included. | Results revised. |
| Figures: ensure maps (Figure 1 and hotspot maps) are high resolution and labeled with clear legends. | Figures have been reviewed again. | Figures reviewed. |
| Expand on policy implications: how can SUS or municipal governments use these findings? | Discussion expanded accordingly. | Discussion revised. |
| Compare internationally. | All possible international comparisons have been incorporated. | Discussion revised. |
| Expand limitations. | Additional limitations have been discussed. | Discussion revised. |
| Correct typos and terminology. | Corrected throughout the manuscript. | Entire manuscript revised. |
| Remove redundancy. | Redundant wording has been reduced. | Entire manuscript revised. |

**Table 3. Response to Reviewer #2**

| **Reviewer Comment** | **Author Response** | **Changes Made** |
| --- | --- | --- |
| Justify spatial analysis. | Thank you for this important observation. The Introduction has been expanded to better explain why delayed healthcare access may exhibit geographic variation and why spatial analysis is appropriate for identifying spatial inequalities in healthcare access. | Introduction revised. |
| Explain geographic variation. | Additional discussion has been added describing contextual and structural factors that may contribute to geographic variation. | Introduction revised. |
| Demonstrate spatial heterogeneity. | Global Moran's I analysis was conducted prior to hotspot analysis and is now presented as evidence supporting spatial heterogeneity. | Results revised. |
| Explain analytical framework. | The analytical framework has been reorganized to present Moran's I, hotspot analysis, multilevel analysis, and spatial modelling in a logical sequence. | Methods revised. |
| Consider spatial regression. | We appreciate this important methodological point. We recognize that while significant spatial clustering was detected (Moran's I p < 0.001), we did not apply spatial regression models such as spatial lag or spatial error models. However, we used a two-level mixed-effects logistic regression model with municipality-level random effects, which addresses the hierarchical structure of the data and accounts for unobserved heterogeneity between municipalities. This approach is methodologically appropriate for nested data structures (individuals within municipalities) and, combined with our spatial analysis, provides a comprehensive analytical framework. We acknowledge this as a limitation and have added a discussion point suggesting that future studies could extend this work by applying spatial regression models (e.g., spatial lag, spatial error, or geographically weighted regression) to further refine the estimates of contextual effects. | Added acknowledgment and future research direction in the **Limitations** section: "While we used multilevel modeling to account for the hierarchical structure of the data and spatial methods to detect clustering, we did not apply spatial regression models such as spatial lag or spatial error models. Future studies could extend this work by applying geographically weighted regression (GWR) or Bayesian spatial models to further refine the estimates of contextual effects and explicitly model spatial dependence in the regression coefficients." |
| Furthermore, the authors should evaluate key assumptions underlying spatial analyses, including spatial dependence, spatial stationarity versus non-stationarity, and residual spatial autocorrelation. Incorporating these analyses would substantially strengthen the methodological rigor of the study and improve the validity and interpretation of the findings | Additional methodological details regarding spatial weights, residual spatial autocorrelation, and assumptions have been included. | Methods revised. |
