# Supplementary material for: Spatial and multilevel determinants of delayed healthcare access following occupational accidents in Brazil
Source: PLoS One. 2026 Oct 23;21(10):e0360805. doi: 10.1371/journal.pone.0360805 (PMC13693904; doi:10.1371/journal.pone.0360805)
Supplement: Peer review history

# Peer review: Decision Letter 0

**DOI:** 10.1371/journal.pone.0360805.r001

**Authors:** Sandra Maria Oliveira

20 Jul 2026

Dear Dr. Debalkie Animut,

Thank you for submitting your manuscript to PLOS ONE. After careful consideration, we feel that it has merit but does not fully meet PLOS ONE’s publication criteria as it currently stands. Therefore, we invite you to submit a revised version of the manuscript that addresses the points raised during the review process.

Please submit your revised manuscript by Sep 18 2026 11:59PM. If you will need more time than this to complete your revisions, please reply to this message or contact the journal office at plosone@plos.org. When you're ready to submit your revision, log on to https://www.editorialmanager.com/pone/ and select the 'Submissions Needing Revision' folder to locate your manuscript file.

- A letter that responds to each point raised by the academic editor and reviewer(s). You should upload this letter as a separate file labeled 'Response to Reviewers'.
- A marked-up copy of your manuscript that highlights changes made to the original version. You should upload this as a separate file labeled 'Revised Manuscript with Track Changes'.
- An unmarked version of your revised paper without tracked changes. You should upload this as a separate file labeled 'Manuscript'.

If you would like to make changes to your financial disclosure, please include your updated statement in your cover letter. Guidelines for resubmitting your figure files are available below the reviewer comments at the end of this letter.

If applicable, we recommend that you deposit your laboratory protocols in protocols.io to enhance the reproducibility of your results. Protocols.io assigns your protocol its own identifier (DOI) so that it can be cited independently in the future. For instructions see: https://journals.plos.org/plosone/s/submission-guidelines#loc-laboratory-protocols. Additionally, PLOS ONE offers an option for publishing peer-reviewed Lab Protocol articles, which describe protocols hosted on protocols.io. Read more information on sharing protocols at https://plos.org/protocols?utm_medium=editorial-email&utm_source=authorletters&utm_campaign=protocols.

As the corresponding author, your ORCID iD is verified in the submission system and will appear in the published article. PLOS supports the use of ORCID, and we encourage all coauthors to register for an ORCID iD and use it as well. Please encourage your coauthors to verify their ORCID iD within the submission system before final acceptance, as unverified ORCID iDs will not appear in the published article. *Only*  the individual author can complete the verification step; PLOS staff *cannot*  verify ORCID iDs on behalf of authors.

We look forward to receiving your revised manuscript.

Kind regards,

Sandra Maria Valle Leone de Oliveira

Academic Editor

PLOS One

Journal Requirements:

When submitting your revision, we need you to address these additional requirements.

1. Please ensure that your manuscript meets PLOS ONE's style requirements, including those for file naming. The PLOS ONE style templates can be found at

https://journals.plos.org/plosone/s/file?id=wjVg/PLOSOne_formatting_sample_main_body.pdf and https://journals.plos.org/plosone/s/file?id=ba62/PLOSOne_formatting_sample_title_authors_affiliations.pdf

2. Your ethics statement should only appear in the Methods section of your manuscript. If your ethics statement is written in any section besides the Methods, please move it to the Methods section and delete it from any other section. Please ensure that your ethics statement is included in your manuscript, as the ethics statement entered into the online submission form will not be published alongside your manuscript.

3. In the online submission form, you indicated that the data that support the findings of this study are available from the corresponding author upon reasonable request.

All PLOS journals now require all data underlying the findings described in their manuscript to be freely available to other researchers, either 1. In a public repository, 2. Within the manuscript itself, or 3. Uploaded as supplementary information.

This policy applies to all data except where public deposition would breach compliance with the protocol approved by your research ethics board. If your data cannot be made publicly available for ethical or legal reasons (e.g., public availability would compromise patient privacy), please explain your reasons on resubmission and your exemption request will be escalated for approval.

4. We note that Figure(s) 2, 3, 4 in your submission contain [map/satellite] images which may be copyrighted. All PLOS content is published under the Creative Commons Attribution License (CC BY 4.0), which means that the manuscript, images, and Supporting Information files will be freely available online, and any third party is permitted to access, download, copy, distribute, and use these materials in any way, even commercially, with proper attribution. For these reasons, we cannot publish previously copyrighted maps or satellite images created using proprietary data, such as Google software (Google Maps, Street View, and Earth). For more information, see our copyright guidelines: http://journals.plos.org/plosone/s/licenses-and-copyright.

We require you to either (1) present written permission from the copyright holder to publish these figures specifically under the CC BY 4.0 license, or (2) remove the figures from your submission:

a. You may seek permission from the original copyright holder of Figure(s) 2, 3, 4 to publish the content specifically under the CC BY 4.0 license.

We recommend that you contact the original copyright holder with the Content Permission Form (http://journals.plos.org/plosone/s/file?id=7c09/content-permission-form.pdf) and the following text:

“I request permission for the open-access journal PLOS ONE to publish XXX under the Creative Commons Attribution License (CCAL) CC BY 4.0 (http://creativecommons.org/licenses/by/4.0/). Please be aware that this license allows unrestricted use and distribution, even commercially, by third parties. Please reply and provide explicit written permission to publish XXX under a CC BY license and complete the attached form.”

Please upload the completed Content Permission Form or other proof of granted permissions as an "Other" file with your submission.

In the figure caption of the copyrighted figure, please include the following text: “Reprinted from [ref] under a CC BY license, with permission from [name of publisher], original copyright [original copyright year].”

b. If you are unable to obtain permission from the original copyright holder to publish these figures under the CC BY 4.0 license or if the copyright holder’s requirements are incompatible with the CC BY 4.0 license, please either i) remove the figure or ii) supply a replacement figure that complies with the CC BY 4.0 license. Please check copyright information on all replacement figures and update the figure caption with source information. If applicable, please specify in the figure caption text when a figure is similar but not identical to the original image and is therefore for illustrative purposes only.

The following resources for replacing copyrighted map figures may be helpful:

USGS National Map Viewer (public domain): http://viewer.nationalmap.gov/viewer/

The Gateway to Astronaut Photography of Earth (public domain): http://eol.jsc.nasa.gov/sseop/clickmap/

Maps at the CIA (public domain): https://www.cia.gov/library/publications/the-world-factbook/index.html and https://www.cia.gov/library/publications/cia-maps-publications/index.html

NASA Earth Observatory (public domain): http://earthobservatory.nasa.gov/

Landsat: http://landsat.visibleearth.nasa.gov/

USGS EROS (Earth Resources Observatory and Science (EROS) Center) (public domain): http://eros.usgs.gov/#

Natural Earth (public domain): http://www.naturalearthdata.com/

5. Please ensure that you refer to Figure 1, 2, 3, 4 in your text as, if accepted, production will need this reference to link the reader to the figure.

6. Please include a separate caption for each figure in your manuscript.

7. If the reviewer comments include a recommendation to cite specific previously published works, please review and evaluate these publications to determine whether they are relevant and should be cited. There is no requirement to cite these works unless the editor has indicated otherwise.

Additional Editor Comments :

The manuscript is relevant and methodologically consistent, however, several issues should be clarified before publication. The statistical analysis is appropriate, although it is important to verify and report any residual spatial autocorrelation after the multilevel model. Data availability should also be clarified to ensure compliance with the PLOS ONE data-sharing policy. The English is generally clear and understandable, requiring only minor language and stylistic revisions.

It is recommended to revise the definition of the outcome, as the term delayed healthcare seeking may imply an individual decision, whereas delays exceeding 24 hours may also reflect geographic, organizational, and structural barriers to healthcare access. The rationale for using a 24-hour cutoff should be explicitly justified.

The justification for the spatial analysis should be strengthened by providing a clearer explanation of the spatial weights matrix, the criteria used to identify hotspots, and the assessment of residual spatial dependence. As this is a cross-sectional study, causal terms such as determinants should be replaced with associated factors, and definitive causal statements should be moderated.

The policy recommendations should be consistent with the study findings and clearly distinguished from proposals that were not directly evaluated. In addition, the reported prevalence of 12.2% should not be generalized to all Brazilian workers, as the data are limited to cases reported and included in the SINAN database.

Finally, the discussion of limitations should be expanded to address potential underreporting, data incompleteness, and regional differences in surveillance quality. The interpretation of the findings should emphasize the magnitude and epidemiological relevance of the observed effects, rather than relying solely on statistical significance.

[Note: HTML markup is below. Please do not edit.]

Reviewers' comments:

Reviewer's Responses to Questions

**Comments to the Author**

1. Is the manuscript technically sound, and do the data support the conclusions?

Reviewer #1: Yes

Reviewer #2: Yes

**********

2. Has the statistical analysis been performed appropriately and rigorously?

Reviewer #1: Yes

Reviewer #2: No

**********

3. Have the authors made all data underlying the findings in their manuscript fully available?

The PLOS Data policy

Reviewer #1: No

Reviewer #2: Yes

**********

4. Is the manuscript presented in an intelligible fashion and written in standard English?

Reviewer #1: Yes

Reviewer #2: Yes

**********

Reviewer #1: Abstract

Strengths: Clear statement of background, methods, and key findings. The prevalence (12.2%) and spatial clustering results are well highlighted.

Suggestions:

o The abstract is slightly dense; consider shortening methodological detail (e.g., “Global Moran’s I and Getis–Ord Gi* statistics” could be summarized as “spatial clustering techniques”).

o Emphasize the policy relevance more strongly in the conclusion (e.g., “Findings highlight urgent need for geographically targeted occupational health interventions”)

Introduction

• Strengths: Excellent contextualization with global and national statistics. The use of the “Three Delays Model” is appropriate and strengthens theoretical framing.

• Suggestions:

o Some sentences are long and could be split for readability (e.g., “Beyond the human toll, OA exacts massive socioeconomic consequences…”).

o Clarify novelty: explicitly state how this study differs from prior SINAN based analyses (e.g., “Unlike previous descriptive studies, this research integrates multilevel and spatial modeling”).

o Consider tightening references: currently, citations (5, 6, 8, 9, 13, 15–17) are clustered; grouping them by theme would improve flow

Methods

• Strengths: Very thorough description of data cleaning, variable definitions, and statistical modeling. The inclusion of ICC, MOR, and PCV adds robustness.

• Suggestions:

o The statistical model equation is well presented, but could be simplified for non technical readers (perhaps move full formula to appendix).

o Clarify exclusion criteria: specify how many records were removed at each step (duplicates, missing values, negative time differences).

o Spatial analysis: explain why inverse distance weighting was chosen over contiguity matrices.

Results

• Strengths: Tables are clear, with large sample sizes well summarized. Regional and occupational breakdowns are informative.

• Suggestions:

o Tables 1–3 are heavy with numbers; consider adding plain language summaries (e.g., “Most injuries occurred among men in their 20s and 30s, particularly in manual/industrial occupations”).

o Highlight effect sizes more narratively: e.g., “Evening injuries were over three times more likely to result in delayed care.”

o Figures: ensure maps (Figure 1 and hotspot maps) are high resolution and labeled with clear legends.

Discussion

• Strengths: Strong linkage between findings and structural inequalities in Brazil’s health system.

• Suggestions:

o Expand on policy implications: how can SUS or municipal governments use these findings?

o Compare with international evidence: are similar spatial disparities seen in other LMICs?

o Acknowledge limitations more explicitly (e.g., reliance on secondary data, potential underreporting in SINAN, exclusion of informal accidents not reported)

Language & Style

• Generally formal and precise, but a few issues:

o Minor typos: “Corrosponding author” → “Corresponding author.”

o Consistency: use “Delayed healthcare access” or “Delayed healthcare seeking” consistently.

o Avoid redundancy: “municipal-level contextual variables” is repeated several times.

Overall Assessment

This is a strong manuscript with robust methodology and clear findings. To maximize impact:

• Streamline abstract and introduction.

• Add narrative summaries to results.

• Strengthen discussion with policy recommendations and international comparisons.

• Ensure figures and tables are visually accessible.

Reviewer #2: The manuscript addresses an important public health problem: delayed healthcare access following occupational injury which has significant implications for injury management, disability prevention, and health equity. The study is relevant and has the potential to contribute meaningfully to occupational health research.

However, the rationale for conducting a spatial analysis is not sufficiently developed. The authors should clearly explain why a spatial approach is appropriate for this research. Specifically, they should articulate whether delayed healthcare access following occupational injury varies geographically across Brazil and identify the contextual or geographic factors that may explain these spatial variations. Establishing the presence of spatial heterogeneity is essential to justify the use of spatial analytical methods.

If the objective is to investigate geographic patterns, the analysis should follow a logical spatial analytical framework. The authors should first assess the presence of spatial autocorrelation and identify hotspots or clusters (e.g., using Global Moran's I and Local Indicators of Spatial Association [LISA] or Getis-Ord Gi* statistics). If significant spatial clustering is detected, conventional regression models may violate the assumption of independent observations. In that case, spatial regression approaches—such as spatial lag or spatial error models, geographically weighted regression (GWR), multiscale geographically weighted regression (MGWR), or Bayesian spatial models, as appropriate—would provide more valid estimates than classical regression models.

Furthermore, the authors should evaluate key assumptions underlying spatial analyses, including spatial dependence, spatial stationarity versus non-stationarity, and residual spatial autocorrelation. Incorporating these analyses would substantially strengthen the methodological rigor of the study and improve the validity and interpretation of the findings..

**********

what does this mean?). If published, this will include your full peer review and any attached files.

If you choose “no”, your identity will remain anonymous but your review may still be made public.

**Do you want your identity to be public for this peer review?**  For information about this choice, including consent withdrawal, please see our Privacy Policy

Reviewer #1: **Yes:**  Girum Taye Zeleke

Reviewer #2: No

**********

[NOTE: If reviewer comments were submitted as an attachment file, they will be attached to this email and accessible via the submission site. Please log into your account, locate the manuscript record, and check for the action link "View Attachments". If this link does not appear, there are no attachment files.]

To ensure your figures meet our technical requirements, please review our figure guidelines: https://journals.plos.org/plosone/s/figures

You may also use PLOS’s free figure tool, NAAS, to help you prepare publication quality figures: https://journals.plos.org/plosone/s/figures#loc-tools-for-figure-preparation.

NAAS will assess whether your figures meet our technical requirements by comparing each figure against our figure specifications.

# Author comment: Author response to Decision Letter 1

**DOI:** 10.1371/journal.pone.0360805.r002

30 Jul 2026

Dear Editor and Reviewers,

We sincerely thank you for the time and effort invested in reviewing our manuscript entitled "Spatial and multilevel determinants of delayed health care after occupational accidents in Brazil" (PONE-D-26-28045R1). We greatly appreciate the valuable comments and constructive suggestions provided, which have significantly helped us improve the quality and clarity of our work.

We have carefully considered each point raised by the reviewers and have explicitly addressed all comments in detail. Below, we provide a point-by-point response to each reviewer's feedback. All changes made in the revised manuscript have been clearly marked using track changes for easy reference.

Attachment Submitted filename: Response to Reviewers.docx

# Peer review: Decision Letter 1

**DOI:** 10.1371/journal.pone.0360805.r003

**Authors:** Marianne Clemence

2 Oct 2026

Spatial and multilevel determinants of delayed healthcare access following occupational accidents in Brazil

PONE-D-26-28045R1

Dear Dr. Debalkie Animut,

We’re pleased to inform you that your manuscript has been judged scientifically suitable for publication and will be formally accepted for publication once it meets all outstanding technical requirements.

Within one week, you’ll receive an e-mail detailing the required amendments. When these have been addressed, you’ll receive a formal acceptance letter and your manuscript will be scheduled for publication.

An invoice will be generated when your article is formally accepted. Please note, if your institution has a publishing partnership with PLOS and your article meets the relevant criteria, all or part of your publication costs will be covered. Please make sure your user information is up-to-date by logging into Editorial Manager at Editorial Manager® and clicking the ‘Update My Information' link at the top of the page. For questions related to billing, please contact billing support.

If your institution or institutions have a press office, please notify them about your upcoming paper to help maximize its impact. If they’ll be preparing press materials, please inform our press team as soon as possible -- no later than 48 hours after receiving the formal acceptance. Your manuscript will remain under strict press embargo until 2 pm Eastern Time on the date of publication. For more information, please contact onepress@plos.org.

Kind regards,

Marianne Clemence

Staff Editor

PLOS One

Additional Editor Comments (optional):

Reviewers' comments:

Reviewer's Responses to Questions

**Comments to the Author**

Reviewer #1: All comments have been addressed

**********

2. Is the manuscript technically sound, and do the data support the conclusions?

Reviewer #1: Yes

**********

3. Has the statistical analysis been performed appropriately and rigorously?

Reviewer #1: Yes

**********

4. Have the authors made all data underlying the findings in their manuscript fully available?

The PLOS Data policy

Reviewer #1: Yes

**********

5. Is the manuscript presented in an intelligible fashion and written in standard English?

Reviewer #1: Yes

**********

Reviewer #1: The authors have thoroughly addressed all of my previous comments, and the manuscript tackles a timely public health issue of considerable importance. The study is technically sound, with the data clearly supporting the conclusions drawn. Furthermore, the statistical analysis has been conducted appropriately, ensuring the robustness and reliability of the findings.

**********

what does this mean?). If published, this will include your full peer review and any attached files.

If you choose “no”, your identity will remain anonymous but your review may still be made public.

**Do you want your identity to be public for this peer review?**  For information about this choice, including consent withdrawal, please see our Privacy Policy

Reviewer #1: No

**********

# Editor report: Acceptance letter

**DOI:** 10.1371/journal.pone.0360805.r004

**Authors:** Marianne Clemence

PONE-D-26-28045R1

PLOS One

Dear Dr. Debalkie Animut,

I'm pleased to inform you that your manuscript has been deemed suitable for publication in PLOS One. Congratulations! Your manuscript is now being handed over to our production team.

At this stage, our production department will prepare your paper for publication. This includes ensuring the following:

* All references, tables, and figures are properly cited

* All relevant supporting information is included in the manuscript submission,

* There are no issues that prevent the paper from being properly typeset

You will receive further instructions from the production team, including instructions on how to review your proof when it is ready. Please keep in mind that we are working through a large volume of accepted articles, so please give us a few days to review your paper and let you know the next and final steps.

Lastly, if your institution or institutions have a press office, please let them know about your upcoming paper now to help maximize its impact. If they'll be preparing press materials, please inform our press team within the next 48 hours. Your manuscript will remain under strict press embargo until 2 pm Eastern Time on the date of publication. For more information, please contact onepress@plos.org.

You will receive an invoice from PLOS for your publication fee after your manuscript has reached the completed accept phase. If you receive an email requesting payment before acceptance or for any other service, this may be a phishing scheme. Learn how to identify phishing emails and protect your accounts at https://explore.plos.org/phishing.

If we can help with anything else, please email us at customercare@plos.org.

Thank you for submitting your work to PLOS One and supporting open access.

Kind regards,

PLOS One Editorial Office Staff

on behalf of

Dr Marianne Clemence

Staff Editor

PLOS One
